# New methods for estimating follow-up rates in cohort studies

**DOI:** 10.1186/s12874-017-0436-z

**Published:** 2017-12-01

**Authors:** Xiaonan Xue, Ilir Agalliu, Mimi Y. Kim, Tao Wang, Juan Lin, Reza Ghavamian, Howard D. Strickler

**Affiliations:** 1 Department of Epidemiology & Population Health, Albert Einstein College of Medicine, Bronx, NY 10461 USA; 2 Department of Urology, Albert Einstein College of Medicine and Montefiore Medical Center, Bronx, NY 10461 USA

**Keywords:** Person-time, Loss to follow-up, Median survival time, Reverse Kaplan-Meier survival curve, Competing risk

## Abstract

**Background:**

The follow-up rate, a standard index of the completeness of follow-up, is important for assessing the validity of a cohort study. A common method for estimating the follow-up rate, the “Percentage Method”, defined as the fraction of all enrollees who developed the event of interest or had complete follow-up, can severely underestimate the degree of follow-up. Alternatively, the median follow-up time does not indicate the completeness of follow-up, and the reverse Kaplan-Meier based method and Clark’s Completeness Index (CCI) also have limitations.

**Methods:**

We propose a new definition for the follow-up rate, the Person-Time Follow-up Rate (PTFR), which is the observed person-time divided by total person-time assuming no dropouts. The PTFR cannot be calculated directly since the event times for dropouts are not observed. Therefore, two estimation methods are proposed: a formal person-time method (FPT) in which the expected total follow-up time is calculated using the event rate estimated from the observed data, and a simplified person-time method (SPT) that avoids estimation of the event rate by assigning full follow-up time to all events. Simulations were conducted to measure the accuracy of each method, and each method was applied to a prostate cancer recurrence study dataset.

**Results:**

Simulation results showed that the FPT has the highest accuracy overall. In most situations, the computationally simpler SPT and CCI methods are only slightly biased. When applied to a retrospective cohort study of cancer recurrence, the FPT, CCI and SPT showed substantially greater 5-year follow-up than the Percentage Method (92%, 92% and 93% vs 68%).

**Conclusions:**

The Person-time methods correct a systematic error in the standard Percentage Method for calculating follow-up rates. The easy to use SPT and CCI methods can be used in tandem to obtain an accurate and tight interval for PTFR. However, the FPT is recommended when event rates and dropout rates are high.

**Electronic supplementary material:**

The online version of this article (10.1186/s12874-017-0436-z) contains supplementary material, which is available to authorized users.

## Background

The follow-up rate, a standard index of the completeness of follow-up, is important for assessing the adequacy of a prospective or retrospective longitudinal cohort dataset for research purposes. In particular, a low follow-up rate raises concerns regarding the possibility of informative censoring, bias and diminishing statistical power [1–5]; concerns that increase incrementally with the extent of participant dropout from the cohort [5–12]. Common sources of “loss-to-follow-up” include, death due to causes other than the endpoint of interest, patient withdrawal, as well as other reasons for dropout, such as a change in at-risk status (e.g., undergoing a hysterectomy during a study of cervical cancer). For simplicity, in this paper we refer to all loss-to-follow-up and censoring due to any causes other than the event of interest or the end of the study as dropout.

Methods to accurately assess follow-up rates are likely to be of growing importance during the current, expanding era of electronic medical records (EMRs). That is, hospital and outpatient databases are increasingly being exploited for research purposes, but require careful scrutiny to determine whether they are truly adequate for use in scientific studies. Patients in routine clinical practice may be more likely than research volunteers in a prospective cohort to seek care from multiple, unaffiliated providers, leading to low follow-up rates observed at a specific health care facility, raising particular concerns regarding informative censoring. Investigators may therefore need to screen through multiple potential clinics or other sources of EMR data to find an appropriate population with adequate follow-up data.

Thus, while there are many sources of potential bias, the follow-up rate provides a quick and easy tool to initially screen potential retrospective clinical cohorts prior to doing more in depth evaluation of the adequacy of the data. Both the researchers and journal reviewers should therefore routinely examine the follow-up rate in an EMR-based study over a period of observation relevant to the study question.

The most commonly used method to assess the completeness of the follow-up, recommended by Cochrane Handbook [13] and the CONSORT guidelines [14] and often referred to as the “Percentage Method” [15], involves simply calculating the proportion of subjects present at baseline (e.g., enrollment) who remained through the end of the study interval or developed the event of interest by the end of the interval [7, 13, 14, 16]. However, this definition is “naïve” in that it does not distinguish subjects who dropped out early during a study from subjects who dropped out late in the study. In fact, the Percentage Method essentially assumes that all the subjects who were lost to follow-up were lost at the very beginning of the study, and therefore can severely underestimate the follow-up rate in a cohort, leading to a false conclusion regarding the quality of the data.

Several attempts have been made to improve upon the Percentage Method for assessing the degree of follow-up. For example, the median follow-up time has been used as a measure to examine the length of follow-up. However, there have been disagreements regarding how the median follow-up time should be calculated: whether it should be calculated among all subjects, only dropouts, or other variations, each has its limitations [17–20]. Further, there is an increasing recognition that the median follow-up time does not directly measure the “completeness of the follow-up”: e.g., the median follow-up can be low with excellent follow-up, and it can be high with poor follow-up [18, 20–22]. While time to event studies must have sufficient length of follow-up to capture enough events in order to have sufficient statistical power, as we mentioned earlier, poor follow-up raises concern on the validity of the study. Thus, to assess adequate of follow-up for a cohort study, we need to examine both the length and the completeness of follow-up.

Alternatively, a reverse Kaplan-Meier (KM) survival curve has also been used to assess the length as well as the completeness of the follow-up, which is constructed by reversing “censor” and “event” [18]. However, as explained in detail below, because the reverse KM method treats the events of interest as censoring, it exaggerates the cumulative loss to follow-up rate. In addition, a measure of follow-up completeness proposed by Clark et al. [21], which we explained more later, fails to account for possible events that could have occurred among those who were lost to follow-up if they had remained in the study. Further, the accuracy of this method, to our knowledge, was never formally examined using simulations.

In this paper, we review major existing methods for estimating follow-up, and propose a new person-time follow-up rate (PTFR) – essentially, the observed person-time divided by the person-time assuming no dropouts – to address the limitations we found with existing methods. We then describe two methods to estimate PTFR. Simulation studies are used to examine the accuracy of the proposed methods and the existing methods, and each method is applied to a real-world prostate cancer recurrence “retrospective cohort” study based on EMR data [23].

### Existing measures for following-up rates

Consider a cohort of size N, and that T_i_ and C_i_ represent the time to the development of event of interest and the censoring time for the ith subject, respectively, *i* = 1,2,…,N. For simplicity, we assume the study ends at a specified time,*τ*.

#### Standard “percentage method”

The Percentage Method *η*_*percentage*_ defines the follow-up rate as

1 $$ {\eta}_{percentage}=\frac{\mathrm{N}\hbox{-} \#\mathrm{lost}\  \mathrm{to}\  \mathrm{follow}\hbox{-} \mathrm{up}}{\mathrm{N}}=\frac{N-\sum \limits_{i=1}^NI\left({T}_i>{C}_i\&{C}_i<\tau \right)}{N}\ast 100\%. $$

**Fig. 1:**
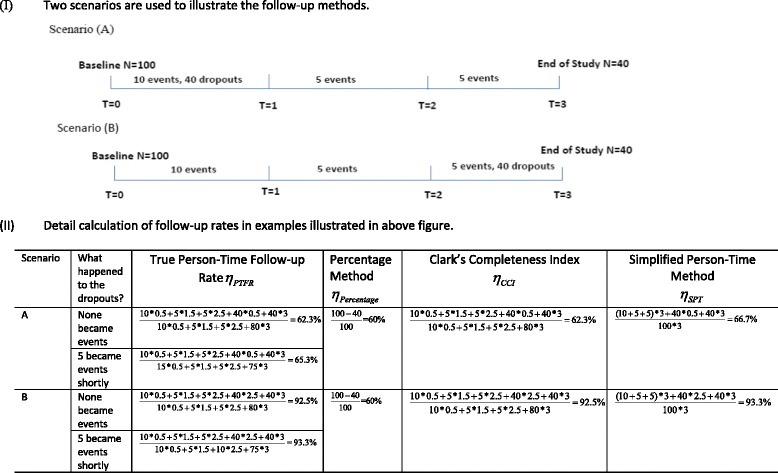
Illustration of the differences in estimates of follow-up using existing and proposed methods. The figure depicts a hypothetical cohort of 100 subjects who were followed and assessed with annual visits for three years. There were 10, 5 and 5 outcome events in the 1st, 2nd and 3rd, respectively. There were 40 dropouts in the 1st year in scenario (A) and in the 3rd year in scenario (B). For simplicity, in this example all events and dropouts occurred on average at the middle of the year. Because the calculation of the true person-time follow-up rate requires the knowledge of the event time for dropouts, we further assumed two situations for the 40 dropouts: (1) none of them became events during the study and (2) 5 of them became events shortly after they dropped out. The Percentage Method (see Eq. (1)) estimates follow-up as the same in both scenarios, since it does not account for person-time in a cohort, and in essence assumes that all dropout occurs at the beginning of the study. Conversely, the Clark Completeness Index (see Eq. (2)) and the Simplified Person-Time Method (see Eq. (5)) both address person-time and provide accurate estimates of the True Person-Time Follow-up Rate (see Eq. (3)). The calculations for each method are shown based on the data from the two scenarios depicted above

In brief, this method calculates the fraction of all enrollees who either developed the outcome of interest or were censored at *τ*. Note that although participants dropped out at different times, the percentage method essentially considers their follow-up time as zero no matter how long they contributed person-time to the study-systematically underestimating the true follow-up. To help illustrate these points, Fig. 1 provides a simple example of a hypothetical cohort of 100 subjects who were followed and assessed with annual visits for three years. There were 10, 5 and 5 outcome events in the 1st, 2nd and 3rd year, respectively with 40 dropouts in the 1st year in scenario (A) and in the 3rd year in scenario (B). The Percentage Method estimates follow-up rate to be 60%, regardless of whether the dropouts occurred at the beginning of the study or late in the study.

As mentioned above, alternative methods have been developed to address the length of actual observation within a cohort. Two of the most commonly referenced are the reverse KM Survival Curve and the Clark et al.’s Completeness Index method [21].

#### Reverse Kaplan-Meier (KM) survival curve

**Fig. 2:**
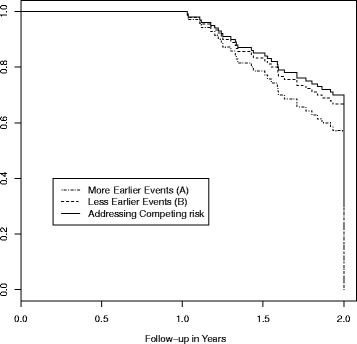
Illustration of the Reverse Kaplan-Meier Survival Curve for follow-up rate. The figure depicts a hypothetical cohort of 100 subjects who were followed for two years, there were 30 outcome events in the 1st year in scenario (A) and 10 outcome events in the 1st year in scenario (B) while in both scenarios there was no dropout in the 1st year and 30 dropouts in the 2nd year. The dashed dotted line describes the reverse KM follow-up rate for scenario (A), the dashed line describes the reverse KM follow-up rate for scenario (B) and the solid line describes the follow-up rate after treating outcome events as competing events. While scenario (A) and (B) have the exactly the same level and timing of dropouts, scenario (A) has a lower follow-up rate simply because it has more earlier events; both scenarios share the same follow-up rate after addressing competing risk. Note: this is not the KM curve for the outcome events. In this plot, losses to follow-up were treated as “events” while development of outcome events were treated as “censored”

The reverse KM survival curve is constructed by reversing “censor” and “event” of the standard KM curve [18]. The advantage of this curve is that it describes the extent as well as the timing of loss to follow-up occurred during the study follow-up. If this curve remained closed to 1 until later in the study, then one can infer nearly complete early follow-up therefore more reliable survival estimates at earlier times than later. However, an important limitation to the reverse KM is that it removes events of interest developed during the study from all subsequent risk sets. Thus, studies with a high early event rate can have a low follow-up rate simply due to a smaller risk set. For example, for a hypothetical cohort of 100 subjects who were followed for two years, there were 30 outcome events in the 1st year in scenario (A) and 10 outcome events in the 1st year in scenario (B) while in both scenarios there was no dropout in the 1st year and 30 dropouts in the 2nd year. As indicated in Fig. 2, the reverse Kaplan-Meier Survival curve estimates a higher follow-up rate over time for scenario (B) simply because that Scenario (B) had less earlier events, despite that both scenarios had exactly the same level and timing of dropouts for cohorts of same size at baseline and of same length of follow-up time. Thus, the reverse KM can be very sensitive to earlier events. Another limitation is that the reverse KM survival curve does not provide a summary measure to assess the completeness of the follow-up by the end of the study.

#### Clark’s completeness index (CCI)

Clark et al. [21] proposed a novel measure to assess completeness of follow-up based on person-time of follow-up:

2 $$ {\eta}_{CCI}=\frac{\ {\mathrm{PT}}_{\mathrm{observed}}\kern0.5em }{{\mathrm{PT}}_{\mathrm{potential}}}=\frac{\sum \limits_{i=1}^N\min \left({T}_i,{C}_i,\tau \right)}{\sum \limits_{i=1}^NI\left({C}_i<\min \left({T}_i,\tau \right)\right)\tau +I\left({C}_i>\min \left({T}_i,\tau \right)\right)\min \left({T}_i,\tau \right)}. $$

Specifically, PT_observed_ = the actual total person-time observed in the study, while PT_potential_ = total potential person-time of follow-up estimated by assuming that all dropouts had the full follow-up time. However, this approach fails to consider that those dropouts could have developed the event of interest during the study interval. Therefore, it can overestimate the total potential follow-up time and consequently underestimate the completeness of follow-up; the extent of underestimation would necessarily increase with higher event and dropout rates. In Fig. 1, *η*_*CCI*_ = 62.3% for scenario (A) and *η*_*CCI*_ = 92.5% for scenario (B), suggesting that the method takes into account observation time for dropouts. However, if in scenario (A) 5 of the 40 dropouts died shortly after dropping out, PT_potential_ would be overestimated and thus *η*_*CCI*_ would underestimate the true follow-up rate. The extent to which this affects the estimates given varying conditions and assumptions, to our knowledge, has not been examined before.

## Methods

### A new person-time definition of follow-up rate (PTFR)

In this paper, we propose a new person-time follow-up rate (PTFR) – essentially, the observed person-time divided by the person-time assuming no dropouts. Specifically, we define the follow-up rate *η*_*PTFR*_ as:

3 $$ {\eta}_{PTFR}=\frac{{\mathrm{PT}}_{\mathrm{observed}}}{{\mathrm{PT}}_{\mathrm{no}\hbox{-} \mathrm{dropout}}}=\frac{\sum \limits_{i=1}^N\min \left({T}_i,{C}_i,\tau \right)}{\sum \limits_{i=1}^N\min \left({T}_i,\tau \right)}\ast 100\% $$

where PT_no-dropout_ = the total person-time that would have been observed in the study if there were no dropouts. The denominator is the hypothetical situation of no dropout, with subjects contributing time to event *T*_*i*_ or time to the end of the study, whichever came first. Note that the calculation of *η*_*PTFR*_ requires that the time to event *T*_*i*_ is known for all participants, whether they dropped out or not.

It can be shown that *η*_*CCI*_ underestimates *η*_*PTFR*_ since

$$ {\eta}_{PTFR}-{\eta}_{CCI}=\frac{\sum \limits_{i=1}^NI\left({C}_i\le {W}_i\right)\left(\tau -{W}_i\right)\sum \limits_{i=1}^N\min \left({\mathrm{C}}_i,{W}_i\right)}{\left(\sum \limits_{i=1}^NI\left({C}_i\le {W}_i\right)\tau +I\left({C}_i>{W}_i\right){\mathrm{W}}_i\right)\sum \limits_{i=1}^N{W}_i}\ge 0 $$

as *W*_*i*_ follows the distribution of *T*_*i*_ truncated at *τ*. Using the example in Fig. 1, if none of the dropouts became events during the study, *η*_*PT*_ = 62.3% for scenario (A) and *η*_*PT*_ = 92.4% for scenario (B), *η*_*PTFR*_ = *η*_*CCI*_; however, if 5 of the dropouts became events shortly after they dropped out, then *η*_*PTFR*_ = 65.3 %  > *η*_*CCI*_.

Because the PTFR cannot be calculated directly since the event times for dropouts are not observed, here we propose two estimation methods.

### A formal method to estimate the person-time follow-up rate (FPT)

We first consider an observational cohort study design that involves repeated serial assessments of participants at fixed time-intervals of equal length (e.g., annual or semi-annual clinical visits). In addition to the baseline visit at t_0_ = 0, we denote the pre-specified visit times as (t_1_, t_2_,…,t_K_) where t_K_ = τ, i.e., the end of the follow-up. It is then assumed that, on average, events and censoring occur midway through each interval, consistent with standard practice in life-table analysis [24]. Therefore, the numerator (i.e., the actual person-time of follow-up) of Eq. (3) is estimated to be

$$ P{\widehat{T}}_{observed}=\sum \limits_{k=1}^K\left({N}_{k-1}-\frac{N_{E_k}+{N}_{C_k}}{2}\right) $$

where *N*_*k* − 1_= number of subjects at risk at the beginning of the time interval k (i.e., at time t_k-1_) and ${N}_k={N}_{k-1}-{N}_{E_k}-{N}_{C_k},{N}_{E_k}$and ${N}_{C_k}$ are number of events and dropouts that occurred during the interval k, respectively.

While PT_observed_ can be easily calculated by summing all participants their observed follow-up time during the study, calculation of the denominator, PT_no-dropout_ in the definition of *η*_*PTFR*_, requires knowledge of the actual time to outcome event for each participant if it happened during the study, regardless whether or not the participant dropped out. This information is typically not available in a real-world study. In an earlier effort to address this problem, Chen, Wei and Huang used the known event rate for the population from which the cohort was derived to calculate “the maximum person-year”, which in our nomenclature, is PT_no-dropout_ [15]. However, it is often difficult to specify the population from which a cohort is derived [25], nor will the event rate be known except for certain general endpoints, such as all-cause mortality. Therefore, this approach is not applicable to most studies.

To estimate PT_no-dropout_, herein we propose estimating the event rate based on the observed data. The survival function and the conditional probability of developing the event of interest are estimated using a nonparametric maximum likelihood approach (NPMLE) proposed by Turnbull [26], equivalent of a Kaplan-Meier survival curve but appropriate for interval observations. To use this approach, all subjects follow-up time need to be described by an interval: if a subject experiences an event between the (k-1)th and kth visit, then that individual’s time to event is described by the interval (t_k-1_,t_k_); if a subject dropped out between the (k-1)th and kth visit, then that individual’s event time is described by an interval (t_k-1_,t_K + 1_) where t_K + 1_ = some large number, such as 100 years(a theoretical time interval that in essence indicates that the person who dropped out will eventually develop an event assuming there are no competing risks); if this subject was free of events till the end of the study t_K_, then that individual is given an interval (t_K_,t_K + 1_). The Interval package in R [27, 28] can be readily applied to estimate the survival curve and the conditional probability of developing the event of interest during each interval.

Next, the expected number of events between (t_k-1_,t_k_) is estimated to be ${N}_{k-1}^{\ast }{\widehat{P}}_k$ where ${N}_{k-1}^{\ast }=$ number of subjects remained in the study at time t_k-1_ if there was no loss of follow-up and ${\widehat{P}}_k=$the estimated conditional probability of event during the kth interval using the NPMLE method for k = 1,…,K and ${N}_0^{\ast }=N$. Therefore, the number of subjects remained in the study at the beginning of the interval k + 1 if there was no loss of follow-up is then ${N}_k^{\ast }={N}_{k-1}^{\ast }-{N}_{k-1}^{\ast }{\widehat{P}}_k$. Then, the expected person time if there was no dropout is estimated to be

$$ P{\widehat{Y}}_{nodropout}=\sum \limits_{k=1}^K\left({N}_{k-1}^{\ast }-\frac{N_{k-1}^{\ast }{\widehat{p}}_k}{2}\right). $$

The Person-time follow-up rate is then estimated to be

4 $$ {\eta}_{FPT}=\frac{{\mathrm{P}\mathrm{T}}_{\mathrm{observed}}}{\widehat{\mathrm{P}}{\mathrm{T}}_{\mathrm{no}\hbox{-} \mathrm{dropout}}} $$

This method, apparently, is relying on the assumption of independent censoring, that is, the event rate of the dropout is the same as that in the general population.

While a prospective epidemiological cohort study may intend to follow participants at serial intervals of approximate equal-length (e.g., annual or semi-annual visits), not every participant returns for each visit or does so at the planned time. This leads to varying lengths of time between visits, which can sometimes be quite extensive. Clinical based cohort studies that involve ad hoc patient follow-up (e.g., cohorts defined retrospectively from hospital EMR) often result in irregular schedules of clinical visits with clustering that does not occur at random (e.g., motivated by symptoms, or an abnormal laboratory test result). To assess the follow-up rate for such data, we extended the proposed approach above to address irregular intervals between visits.

For cohorts involving intermittent and ad hoc follow-up, let $\left({t}_{1_i},{t}_{2_i},\dots, {t}_{K_i}\right)$ be the visit times for the ith person, where K_i_ is either (a) the date of the last visit in the study for the ith person; or (b) the visit that ith person was diagnosed of the event. Then for (a) we used time to the last visit as an estimate of the person’s censoring time, i.e., ${\widehat{C}}_i=\mathrm{m}\widehat{\mathrm{i}}\mathrm{n}\left({\mathrm{T}}_i,{C}_i\right)={t}_{K_i}$, and for (b)we estimate the time to event occurred in the mid of the interval, i.e.,${\widehat{T}}_i=\mathrm{m}\widehat{\mathrm{i}}\mathrm{n}\left({T}_i,{C}_i\right)=\frac{t_{K_i-1}+{t}_{K_i}}{2}$. The actual Person-time of follow-up by a specified time, say, *t*_*K*_, is then estimated by the summation of all the observed follow-up times across subjects, i.e.,

$$ P{\widehat{T}}_{observed}=\sum \limits_{i=1}^NI\left(\min \left({\mathrm{T}}_i,{C}_i\right)<{t}_K\right)\mathrm{m}\widehat{\mathrm{i}}\mathrm{n}\left({T}_i,{C}_i\right)+I\left(\min \left({\mathrm{T}}_i,{C}_i\right)\ge {t}_K\right){\mathrm{t}}_K. $$

To estimate PT_no-dropout_, if the ith person developed the event at his/her last visit, the interval event time is $\left({t}_{K_i-1},{t}_{K_i}\right)$ and if a person did not develop event at his/her last visit, the interval event time is then $\left({\mathrm{t}}_{K_i},\mathrm{E}\right)$ where again E represents some large number. Then the NPMLE method can be applied to PT_no-dropout_.

As mentioned above, the use of observed data to estimate the event rate relies on the assumption that the loss to follow-up is not informative, i.e., event rate among those who remained in the study is the same as those who dropped out so that the event rate estimates obtained from the observed data apply to the unobserved. However, if the subjects who were lost to follow-up are at a different risk of recurrence than those who remained in the study, the estimates of event rates are biased. For example, if the subjects who were lost to follow-up had a higher risk of event, then the event risk is under-estimated using the observed data and the follow-up rate will be underestimated using the person-time approach because *PY*_*nodropout*_ is overestimated. Conversely, if the subjects who were loss to follow-up had a lower risk of event, then the event risk is over-estimated and the follow-up rate will consequently be overestimated using the Person-time approach. Here we proposed to calculate a lower bound to the Person-time follow-up rate by assuming all those who dropped out never developed event of interest during the time interval we examined. In this case, *PY*_*nodropout*_reaches its highest possible value, leading to a lower bound for the follow-up rate. Note in this case *PY*_*nodropout*_ = *PY*_*potential*_ so that min *η*_*PTFR*_ = *η*_*CCI*_. The lower bound of the follow-up rate is important because it provides a conservative estimate of the follow-up rate: if the follow-up rate was over-estimated it can lead to over-optimism on the quality of the follow-up.

### A simplified method to estimate the person-time follow-up rate (SPT)

The need to estimate the event rate for the purpose of calculating the PTFR can be difficult especially to a non-statistician. Therefore, we also explore a simplified alternative method to allow quick estimation of *η*_*PTFR*_ without having to estimate the event rate. Our proposed Simplified Person-Time method is a hybrid method including aspects of the Percentage Method and the Person-Time Method. Specifically, as in the Percentage Method, individuals who developed the event of interest during the study are treated the same as individuals who were followed till the end of the study, i.e., they are treated as having contributed complete follow-up since they have already provided complete data regarding the factors associated with becoming a case. Furthermore, as a Person-Time Method, dropouts contribute partial follow-up time in the numerator.

A simple alternative method to calculate the follow-up rate is therefore

5 $$ {\eta}_{SPT}={\frac{\sum \limits_{i=1}^NI\left({C}_i<\min \left({T}_i,\tau \right)\right){C}_i+I\left({C}_i>\min \left({T}_i,\tau \right)\right)\tau }{N\tau}}^{\ast }100\%. $$

Therefore, in Fig. 1, *η*_*SPT*_ = 66.7% for scenario (A) and *η*_*SPT*_ = 93.3% for scenario (B), remarkably close to but slightly overestimate *η*_*PTFR*_, the slight overestimation is because events are given the full length of follow-up in this method. It can be shown that

$$ {\eta}_{PTFR}-{\eta}_{SPT}\le \frac{\sum \limits_{i=1}^NI\left({C}_i>{W}_i\right)\left({W}_i-\tau \right)}{\sum \limits_{i=1}^N{W}_i}\le 0. $$

Figure 1 also indicated that *η*_*CCI*_ and *η*_*SPT*_ together provides a close boundary for *η*_*PTFR*_. In fact, the outcome events can be viewed as competing risk to loss to follow-up and we can therefore use the method in competing risk framework for the computation of cumulative loss to follow-up rate [29, 30] and then to obtain the subdistribution reverse KM curve.

To revisit the reverse KM survival time, we will instead assign the events to have full follow-up time and then the rate of follow-up over time is no longer affected by the amount and the timing of the events. In Fig. 2, both scenarios (A) and (B) will share the same curve of follow-up rate over time after addressing the competing risk of events. It can be shown mathematically that the area under the curve of this new follow-up rate over time divided by τ is *η*_*SPT*_.

R program for computation of each method is provided in Additional file 1.

### Simulation studies

Simulation studies were used to examine follow-up rates computed using the standard Percentage Method, the CCI, the FPT, and the SPT as compared to the true follow-up rate *η*_*PTFR*_. To conduct these comparisons, we assumed a range of different outcome event rates and dropout rates. Specifically, the simulations involved *N* = 1000 subjects and time-to-event and time-to-dropout were generated for each subject using exponential distributions. The event rate was varied between 5% to 50% and the dropout rate from 10% to 50%, which covers a wide range of plausible values for these two parameters. In the first scenario of the simulation, the length of the study was five years with annual clinical visits; the second scenario incorporated random variations in the time between clinic visits (from 0.5 to 1.5 years). The results were then averaged across 1000 simulated datasets.

### Application to the prostate cancer clinical cohort study

A retrospective clinical cohort study of time to recurrence of prostate cancer (PrCa) was conducted using EMRs among patients who underwent robotic assisted laparoscopic prostatectomy (RALP) by a single surgeon at Montefiore Medical Center in the Bronx from October, 2005 through December, 2012 [23]. We used this dataset as a real-world example with staggered study entry and ad hoc follow-up. The dataset included *N* = 610 PrCa patients. Clinical guidelines held that PrCa patients should have PSA levels measured every 3 to 4 months in the first year following RALP, every 6 months in the second and third year, and then annually. However, PSA measurements were to be conducted more frequently if the post-operative serum PSA value exceeded 0.1 ng/dl. The median number of follow-up serum PSA measurements was 7 (range 1–28). PrCa recurrence was defined as a rise in serum PSA of 0.2 ng/ml or higher. There were 87 (14.3%) recurrence events following RALP. Three-year and five-year recurrence rates were of primary interest.

Note although there were no observed deaths in the study, death can be a potential competing risk here. For the interest of assessing the completeness of the follow-up, death should be included as an event when calculating the follow-up rate.

## Results

### Simulation studies

Table 1 shows that across a wide range of dropout and event rates, *η*_*percentage*_ systematically underestimated the follow-up rate: the larger the dropout rate, the higher the level of underestimation. For example, when the event rate was fixed at 10%, the averaged *η*_*percentage*_ varied from 91.0% to 46.4%, whereas the true *η*_*PTFR*_ varied from 95.3% to 68.4%. In contrast, the FPT *η*_*FPT*_ consistently provided an accurate estimate of *η*_*PTFR*_ with bias less than 2%. The downward bias is because the Turnbull’s NPMLE [26] tends to slightly underestimate the event rate consequently the follow-up rate. This under-estimation of the cumulative incidence function using the NPMLE method for interval-censored data has been recognized [31, 32] and more research on alternative estimators are needed.

**Table 1:** Follow-up rates under varying assumptions estimated using four methods: (i) the standard Percentage Method (Eq. 1), (ii) the Clark’s Completeness Index (CCI, Eq. 2), (iii) the Person-Time Method estimated using the formal method (FPT, Eq. 4) and (iv) the Simplified Person-Time Method (SPT, Eq. 5)

Assumed event rate	True Person-time follow-up rate *η*_*PTFR*_	Percentage Method *η*_*percentage*_			Estimated using the formal method *η*_*FPT*_			Clark’s compleness inex *η*_*CCI*_			Simplified Person-time method *η*_*SPT*_		
		Average	%bias^1^	$\sqrt{\mathrm{MSE}}$ ^2^	Average	%bias	$\sqrt{\mathrm{MSE}}$	Average	%bias	$\sqrt{\mathrm{MSE}}$	Average	%bias	$\sqrt{\mathrm{MSE}}$
5%	95.0%	90.4%	−4.90	.047	95.0%	0.00	.001	94.9%	−0.08	.001	95.1%	0.05	.001
	81.9%	66.7%	−18.5	.158	82.1%	0.16	.002	81.7%	−0.26	.003	82.1%	0.25	.003
	68.1%	44.8%	−34.3	.233	68.3%	0.45	.004	67.9%	−3.49	.003	68.2%	0.59	.005
	56.7%	29.3%	−48.2	.274	57.3%	0.92	.006	56.5%	−0.27	.003	57.3%	1.09	.007
10%	95.2%	91.0%	−4.48	.043	95.2%	−0.01	.002	95.1%	−0.29	.003	95.4%	0.10	.002
	82.1%	67.9%	−17.4	.142	82.3%	0.17	.002	81.6%	−0.69	.006	82.5%	0.49	.006
	68.5%	46.5%	−32.2	.220	68.9%	0.64	.005	67.9%	−1.11	.008	69.2%	1.07	.009
	56.4%	30.3%	−46.2	.261	57.1%	1.33	.008	55.6%	−1.36	.008	57.4%	1.84	.011
30%	94.4%	90.0%	−4.61	.044	93.4%	−0.95	.009	93.7%	−0.64	.006	94.6%	0.31	.004
	82.7%	70.7%	−14.5	.120	82.3%	−0.04	.004	81.1%	−1.94	.016	83.6%	1.06	.009
	69.4%	50.9%	−26.8	.186	69.7%	0.42	.005	67.1%	−3.33	.023	70.9%	2.17	.016
	53.6%	31.0%	−42.2	.226	54.8%	2.17	.012	51.1%	−4.71	.026	55.9%	4.19	.023
50%	93.2%	89.5%	−3.97	.037	88.3%	−0.53	.049	91.2%	−2.08	.020	93.9%	0.80	.008
	77.6%	67.2%	−13.4	.105	74.6%	−3.79	.030	72.5%	−6.58	.051	79.9%	3.00	.024
	65.0%	51.1%	−2.14	.140	63.6%	2.03	.014	58.5%	−9.93	.647	68.4%	5.25	.035
	46.6%	31.3%	−32.8	.153	47.8%	0.03	.014	40.0%	−14.0	.066	51.3%	10.0	.005

These results were compared to the true Person-time follow-up Rate (Eq. 3) based on complete information generated under the simulations, each averaged across 1000 simulated data sets. The simulations involved an assumed 5-year prospective cohort study of *N* = 1000 subjects with fixed annual interval clinical visits and non-informative dropout. Time-to-event was generated based on exponential distributions with event rates varied from 5 to 50% and time to dropout was generated based on an independent exponential distribution with dropout proportion varying from 10 to 70%. Results were averaged across the 1000 simulated datasets

Note: 1. % bias was calculated as (average of the particular method-*η*_*PTFR*_)/*η*_PTFR_*100%; 2. $\sqrt{\mathrm{MSE}}$ was calculated as the square root of the average of (estimate-*η*_*PTFR*_)^2^. MSE from the true *η*_*PTFR*_was calculated instead of variance because several methods used here can be biased

The *η*_*CCI*_ in general provided a good but slightly lower estimate of *η*_*PTFR*_, except when both the event and dropout rates were high because it fails to take into account events occurred in dropouts. For example, when the event rate was 50% and dropout was 70%, the true *η*_*PTFR*_ = 46.6% while *η*_*CCI*_ = 40.0%, a 14% downward bias. The *η*_*SPT*_ is also in close agreement with the true person-time follow-up rate *η*_*PTFR*_ but slightly higher because the events are given the full length of follow-up. The overestimation is also more apparent when the event and dropout rates are high. In the same above example, *η*_*SPT*_ = 51.3%, a 10% upward bias. Careful examination of Table 1 shows that the easily estimable SPT and the CCI were as likely to be the closest to the “True Person-Time” follow-up rate in most scenarios as the more complex and laborious FPT. When *η*_*SPT*_ is used in tandem with *η*_*CCI*_, they provide a tight range of the true follow-up rate so that the use of *η*_*FPT*_ is not necessary.

Similar results for each of the methods of estimating follow-up rates were obtained when visits were irregular; i.e., allowing the time-intervals between visits to vary within a person and between persons (results not shown).

### Example dataset

Table 2 shows the follow-up time as estimated by the Percentage Method, the CCI, the FPT, and the SPT. Because event rates and dropout rates are low, as expected, the FPT, the CCI and the SPT provided similar results. These results provide much higher estimated follow-up than that calculated using the naïve Percentage Method. In fact, had the Percentage Method approach been used, the investigator may have falsely concluded that the dataset had inadequate 5-year follow-up to be suitable for research purposes, when in fact the other methods showed follow-up to be >90% after 5-years.

**Table 2:** The follow-up rate at each annual interval after subjects (*N* = 610) in a retrospective cohort study of 3-year and 5-year prostate cancer (PrCa) recurrence risk based on electronic medical record (EMR) data

Follow-up^b^	N^c^	Percentage Method *η*_*percentage*_	Estimated follow-up using the formal method *η*_*FPT*_	Clark’s completeness index *η*_*CCI*_	Simplified Person-time Method *η*_*SPT*_
1 Year	558	91.4%	95.7%	95.5%	95.7%
2 Year	472	86.2%	95.0%	94.5%	95.0%
3 Year	383	80.9%	93.6%	92.9%	93.8%
4 Year	295	75.6%	92.5%	92.3%	93.3%
5 Year	197	67.5%	91.8%	91.8%	93.0%

Follow-up rates were estimated using four methods, namely, (i) the standard Percentage Method (Eq. 1 ^a^), (ii) the formal Method (FPT, Eq. 4), (iii) the Clark’s completeness index (CCI, Eq. 2), and (iv) the Simplified Person-Time Method (SPT, Eq. 5)

^a^Equations are shown in the next

^b^A retrospective cohort study was conducted among incident PrCa patients who underwent robotic assisted laparoscopic prostatectomy (RALP) by a single surgeon at Montefiore Medical Center (MMC) in the Bronx from 10/2005 through 12/2012. These subjects were followed for disease recurrence or progression through December 2012. A total of N = 610 PrCa patients who underwent RALP and had their follow-up at MMC were included in this analysis

^c^We calculated the follow-up rate at each year among the subset of the patients who had RALP early enough to be eligible for such length of follow-up. For example, to estimate the three year follow-up rate, we calculated this rate among the subjects who had RALP at least before 12/31/2009

In case of informative censoring, as mentioned in the method section, the CCI estimate provides a lower bound for the person-time follow-up rate. Table 2 showed that the lower bounds were very close to the Person-time estimates, suggesting that even in the extreme case that all the dropouts have no risk of developing event during the study, we do not expect the true follow-rate to be much lower.

## Discussion and Conclusion

The completeness of follow-up and the length of follow-up are important measures to determine the adequacy of a cohort dataset for research purposes. The longer the follow-up is, the less the concern regarding statistical power; the better the follow-up is, the less the concern regarding the validity of a study. This paper focused on measures to assess the completeness of the follow-up. A commonly used follow-up rate to assess the completeness of the follow-up, the naïve Percentage Method, fails to consider the person-time contributed to a study by subjects who drop out prior to study completion; other existing measures of completeness of the follow-up including the reverse Kaplan-Meier survival curve and the Clark’s completeness index (CCI) all have its own limitations. Therefore, we define a new follow-up rate based on total observed person-time of follow-up out of the total person-time of follow-up that could have been observed if there was no dropout. This definition corrects the inherited biases in the existing methods.

We next proposed two methods to estimate the proposed Person-Year follow-up rate. In the formal person-time method, we proposed to estimate the event rate using the observed data, based on which we then estimate the expected number of events if they were no dropouts. Note non-informative censoring is assumed for the validity of FPT, that is, event rate among the dropouts is the same as those who did not. Although this assumption is not verifiable, sensitivity analyses can be conducted to examine the robustness of the estimate of the follow-up rate, for example, by assuming that the dropouts have either a higher event rate or lower event rate than those who did not drop out. The second simplified method (SPT) assigns event time as full follow-up therefore does not require the estimation of event rate and consequently is much easier to use.

Our simulations showed that the Percentage Method often underestimates the follow-up rate quite extensively when the dropouts occurred later in the study. The FPT performed well and the CCI and SPT also performed well in most scenarios, while the CCI tends to slightly underestimate and the SPT slightly overestimate the follow-up rate. The bias can be moderate only when both the event rate and the dropout rate are high; otherwise, the SPT used in tandem with the CCI provides an accurate and tight interval estimate of the true Person-time follow-up rate. In these cases, the use of FPT which involves more computations is not necessary. However, the FPT is recommended when event rates and dropout rates are high.

Application of the methods to an example dataset, based on a study of prostate cancer recurrence, helped demonstrate the critical importance of considering person-time prior to dropout when estimating follow-up rates. Briefly, using the standard Percentage Method the 5-year follow-up rate was estimated to be approximately 68%, whereas the CCI, the FPT and SPT all showed the follow-up to be greater than 90%.

Although the CCI method has been proposed over a decade ago, the use of this person-time method to determine follow-up rates has not been widely adopted, likely due to the fact that the performance of the CCI has not been fully examined and/or the misconception that median follow-up time and the reverse KM survival curve are sufficient. Thus, the presentation of this work is timely. The availability and ease of the calculation of the proposed person-time follow-up rate can represent an important advance in assessing the completeness of the follow-up.

Guidelines on how much the extent of loss to follow-up can be problematic have been based primarily on the percentage method. New guidelines that are based on the person-time follow-up rate should be developed to suggest “acceptable” and “alarming” follow-up rates. Recent work by von Allmen [33] examined the bias in estimating mortality rate under various levels of CCI. However, this work did not distinguish missing mechanisms including missing completely at random, missing at random and missing not at random; further, research studies are often interested in obtaining an unbiased estimate of the exposure-disease association or relative risk associated with the exposure instead of absolute risk of death or disease. Therefore, further studies including conducting series of simulation studies to examine the bias and efficiency loss on relative risk estimates under various levels of loss to follow-up measured by our proposed person-time follow-up rates and under various missing mechanisms are needed and will be the primary focus of our future research.

## Additional file

Additional file 1: R program for Computation of the traditional Percentage follow-up rate, Formally estimated Person-Time follow-up rate, Clark’s Completeness Index and Simplified Person-Time follow-up rate. (DOCX 14 kb)

## Abbreviations

CCI: Clark’s completeness index
EMR: Electronic medical records
FPT: formal person-time method
KM: Kaplan-Meier
NPMLE: Nonparametric maximum likelihood approach
PTFR: Person-Time Follow-up Rate
SPT: Simplified person-time method
