# Supplementary material for: New methods for estimating follow-up rates in cohort studies
Source: BMC Med Res Methodol. 2017 Dec 1;17:155. doi: 10.1186/s12874-017-0436-z (PMC5709923; doi:10.1186/s12874-017-0436-z)
Supplement: Additional file 1: — R program for Computation of the traditional Percentage follow-up rate, Formally estimated Person-Time follow-up rate, Clark’s Completeness Index and Simplified Person-Time follow-up rate. (DOCX 14 kb) [file 12874_2017_436_MOESM1_ESM.docx]

**R program for Computation of the traditional Percentage follow-up rate, Formally estimated Person-Time follow-up rate, Clark’s Completeness Index and Simplified Person-Time follow-up rate**

# need the following packages

library(survival)

library(splines)

library(perm)

library(Icens)

library(MLEcens)

library(interval)

# data structure

# assign each participant an interval for their time to event as described in text so that

# tfldata consists of three columns, the first one indicates event status (1=event; 0=censored)

# the next two columns are lower and upper bound of an interval during which the patient either developed the # event of interest or droppout, for example, 100 and 200 (in days from enrollment);

nsubject<-nrow(tfldata)

# define the length of the study by which you want to calculate follow-up rate as endfl (an integer)

# for example, 5 years

endfl<-5

# fit interval censored data to obtain survival estimates

icout<-icfit(Surv(tfldata[,2]/365.25,tfldata[,3]/365.25,type="interval2")~1)

# calculate the expected number of death in each interval

esurv<-getsurv(c(1:endfl),icout)

edeath<-rep(0,endfl)

nnow<-nrow(tfldata)

ss<-1.0

for (j in 1:endfl) {

edeath[j]<-nnow*(ss-esurv[[1]]$S[j])

nnow<-nnow-edeath[j]

ss<-esurv[[1]]$S[j]

}

# to calculate PYobs and PYnoloss

newcensor<-ifelse(tfldata[,1]<1,tfldata[,2],tfldata[,3])/365.25

newsurv<-(tfldata[,2]+tfldata[,3])/2.0/365.25

# define variables

nloss<-rep(0,endfl)

ndeath<-rep(0,endfl)

ncurrent<-nsubject

ncurrentl<-nsubject

ncurnls<-nsubject

ncurls<-nsubject

totalobs<-0

totalmax<-0

totallb<-0

satotal<-0

for ( i in 1:endfl) {

# number of loss to follow-up each year

nloss[i]<-sum(newsurv>newcensor & (i-1)<newcensor & newcensor<i)

# number of death each year

ndeath[i]<-sum(newsurv<newcensor & (i-1)<newsurv & newsurv<i)

# adding total observed person years

totalobs<-totalobs+ncurrent-ndeath[i]-nloss[i]+nloss[i]/2+ndeath[i]/2

# adding the maximum observed person years if no loss follow-up

totalmax<-totalmax+ncurnls-edeath[i]/2

# simplified method gives full follow-up person years to events

satotal<-satotal+ncurls-nloss[i]/2

# total person year that assumes all lost follow-up did not become event

totalc<-totalc+ncurrentl-ndeath[i]/2

# updating for each year

# number of participants remained in each interval

ncurrent<-ncurrent-ndeath[i]-nloss[i]

# number of participants remained in each interval if all losses were followed and they are no events;

ncurrentl<-ncurrentl-ndeath[i]

# number of participants if events are treated as remaining in the study until the end (simple alternative approach)

ncurls<-ncurls-nloss[i]

# number of parcicipants if all losses are followed and they have the same rate of events as the non-losses

ncurnls<-ncurnls-edeath[i] }

# naive percentage method

naiveperc<-(ncurrent+sum(ndeath))/nsubject

# Clark’s complete index

pyc<-totalobs/totalc

# proposed person time method

py<-totalobs/totalmax

#simplified person time method

pys<-satotal/(nsubject*endfl)

#result

cbind(endfl,naiveperc,pyc,py,pys)
